# Mucosomes: Intrinsically Mucoadhesive Glycosylated Mucin Nanoparticles as Multi‐Drug Delivery Platform

**DOI:** 10.1002/adhm.202200340

**Published:** 2022-06-04

**Authors:** Cosmin Butnarasu, Paola Petrini, Francesco Bracotti, Livia Visai, Giuseppe Guagliano, Alessandra Fiorio Pla, Ettore Sansone, Sara Petrillo, Sonja Visentin

**Affiliations:** ^1^ Department of Molecular Biotechnology and Health Science University of Turin via Quarello 15 Torino 10135 Italy; ^2^ Department of Chemistry Materials and Chemical Engineering “Giulio Natta” Politecnico di Milano 20133 Italy; ^3^ Molecular Medicine Department (DMM) Centre for Health Technologies (CHT) UdR INSTM University of Pavia Pavia 27100 Italy; ^4^ Medicina Clinica‐Specialistica UOR5 Laboratorio di Nanotecnologie ICS Maugeri IRCCS Pavia 27100 Italy; ^5^ Department of Life Sciences and Systems Biology University of Torino via Accademia Albertina 13 Torino 10123 Italy

**Keywords:** drug‐delivery, mucin, mucoadhesivity, mucosomes, mucus, nanoparticles, one‐pot

## Abstract

Mucus is a complex barrier for pharmacological treatments and overcoming it is one of the major challenges faced during transmucosal drug delivery. To tackle this issue, a novel class of glycosylated nanoparticles, named “mucosomes,” which are based on the most important protein constituting mucus, the mucin, is introduced. Mucosomes are designed to improve drug absorption and residence time on the mucosal tissues. Mucosomes are produced (150–300 nm), functionalized with glycans, and loaded with the desired drug in a single one‐pot synthetic process and, with this method, a wide range of small and macro molecules can be loaded with different physicochemical properties. Various in vitro models are used to test the mucoadhesive properties of mucosomes. The presence of functional glycans is indicated by the interaction with lectins. Mucosomes are proven to be storable at 4 °C after lyophilization, and administration through a nasal spray does not modify the morphology of the mucosomes. In vitro and in vivo tests indicate mucosomes do not induce adverse effects under the investigated conditions. This study proposes mucosomes as a ground‐breaking nanosystem that can be applied in several pathological contexts, especially in mucus‐related disorders.

## Graphical abstract

This paper introduces mucosomes, an innovative protein‐based drug carrier bioinspired by mucus to combat diseases. Mucosomes loaded with active pharmaceuticals are obtained from mucin, the most important protein of mucus, through a lean one‐pot synthesis. They are naturally mucoadhesive and expose on their surface carbohydrate structures, called glycans, which are important binding sites of bacterial and viral surface proteins.

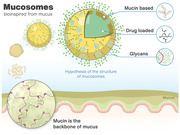

## 1 Introduction

In spite of advances in technology and our knowledge of human diseases, the translation of these benefits into therapeutic advances has been far slower than expected.^[^ ^1^ ^]^ Finding new therapeutic solutions has become increasingly difficult as the cost and time to bring new drugs to market nearly doubled in the last decades, requiring an average investment of $2.6 billion and 12 years of research.^[^ ^2^, ^3^ ^]^ This has, in combination with a high probability of failure during the transition from preclinical studies to clinical trials, made drug development a high‐risk investment. The World Health Organization (WHO) has called for new and more effective strategies to treat the various diseases that take the heaviest toll on the developing world,^[^ ^4^, ^5^ ^]^ yet the current pace of the development of, for example, new antimicrobial drugs is unlikely to meet the challenge of the global emergence of microbial pathogen resistance.^[^ ^6^ ^]^

There is a clear need to reinvent the way in which current drugs are delivered, focusing on different mechanisms of action and improving the efficiency of delivery systems to make them more selective for the specific body district and the type of targeted environment. Within the anti‐infective scenario, for instance, there has been a shift away from destroying the pathogen to interfering with or modulating their pathogenic mechanisms.^[^ ^6^ ^]^ In the anti‐adhesion therapy of infectious agents protein–glycan interactions modulate the adhesion, invasion, and immune system evasion of bacteria and viruses.^[^ ^7^, ^8^, ^9^, ^10^ ^]^ Considering alterations of the glycosylation profile of adhesion‐responsible molecules on the cell surface, such as selectin ligands, integrins, and mucins, protein–glycan interactions result crucial not only for infectious pathogens, but also for their implication in tumor microenvironments:^[^ ^11^, ^12^, ^13^, ^14^ ^]^ an overexpression of sialic acid on the cell surface, for example, increases repulsion between adjacent cells and facilitates entrance into the bloodstream, promoting metastasis.^[^ ^11^, ^15^ ^]^ All of these features could be targeted precisely, switching from generic all‐purpose delivery systems to pathology‐specific or tissue‐specific systems.

The range of nanocarriers, lipid and protein nanoparticles (NPs), currently used to deliver drugs, genetic material or a contrast agent, has clear limitations in terms of safety, efficacy, bioavailability, dose–response, targeting ability, and personalization. A plethora of nanoparticles has been documented with a wide range of applications in medicine and biology and lipid nanoparticles are one of the most preferred platforms for numerous formulations.^[^ ^16^ ^]^ During the last 30 years, their application has been explored and validated in numerous fields, ranging from the administration of pulmonary antibiotic drugs (e.g., Arikayce) to the embedding and delivery of genetic material, including the latest mRNA vaccines against COVID‐19.^[^ ^17^, ^18^ ^]^ Alternatively, it has been demonstrated that protein‐based nanoparticles have advantageous features in the area of oncology: albumin‐bound paclitaxel nanoparticles (nab‐paclitaxel, Abraxane), the only marketed protein‐based nanosystem to date, are used as a second‐line treatment for adult patients with breast metastatic cancer, in combination with gemcitabine, as a first‐line treatment for adult patients with metastatic adenocarcinoma of the pancreas, and, in combination with carboplatin, as a first‐line treatment of non‐small cell lung cancer in adult patients who are not candidates for potentially curative surgery and/or radiation therapy. This pioneering success story provides a glimpse into the opportunities behind this class of nanoparticles as drug vehicles. Several other protein drug carriers, like silk fibroin, gelatin, and gliadin, are also currently under development.^[^ ^19^, ^20^, ^21^ ^]^

With the exception of intravenous administration, all other delivery methods involve overcoming or interacting with physiological barriers to achieve the desired pharmacological effect. The first barrier the between the external environment and all of the wet surfaces of our body is mucus and this substance acts as a primary, innate, defensive barrier since it plays a crucial role in preventing pathogens from reaching the inner areas of the organism, facilitating their expulsion through tightly regulated clearance mechanisms.^[^ ^22^, ^23^ ^]^ Retention of external particles within mucus is mostly governed by the interactions established with mucus components and, in particular, with mucins. Mucins are long polymeric glycoproteins characterized by a peptide backbone which is rich in carbohydrates chains terminated by sialic acid.^[^ ^24^ ^]^ Nevertheless, mucus can equally represent a barrier to overcome when drugs and NPs^[^ ^25^ ^]^ need to be absorbed in mucus‐related disorders characterized by an overproduction of mucus, such as cystic fibrosis (CF), chronic obstructive pulmonary disease (COPD), and bronchial asthma. Often, in these cases, the low efficacy of treatment is the result of the inability of therapeutics to overcome the mucus barrier.^[^ ^26^, ^27^ ^]^

One of the strategies used to improve the performance of pharmaceutical drug formulations involves the adoption of mucoadhesive drug delivery systems. In fact, the adhesion of chemicals to mucous membranes or a mucus‐covered surface prolongs contact with adsorption sites, overcoming the challenge of a short retention time.^[^ ^28^ ^]^ Over the years, several mucoadhesive polymers for drug delivery applications have been investigated. These have included not only natural polymers, such as alginates^[^ ^29^ ^]^ and chitosan,^[^ ^30^ ^]^ but also synthetic such as poly acrylic acid^[^ ^31^ ^]^ and poly vinyl pyrrolidone.^[^ ^32^ ^]^ Developing mucoadhesive drug delivery systems, however, is not straightforward and usually requires ad hoc derivatization procedures which are time consuming and expensive. The pharmaceutical industry, despite the high levels of interest in these solutions, generally adopts less expensive solutions, such as a simple increase in the amount of active ingredient, and has not invested in the designing of mucoadhesive drug delivery systems.^[^ ^33^ ^]^

Acknowledging the need to design drug delivery systems are able to specifically target mucus and carry active pharmaceutical ingredients we have identified an approach which avoids the successive functionalization steps. By mimicking our primary defensive mechanism, the mucus, we used mucin glycoproteins to develop a completely new system with multiple potential applications, including drug delivery, gene‐therapy, and diagnostics. The ability of mucins to directly engage with an extremely wide spectrum of both pathogens and molecules supported our strategy to produce nanosystems for drug delivery, addressing simultaneously the specific challenge of mucoadhesion.

**Figure 1:**
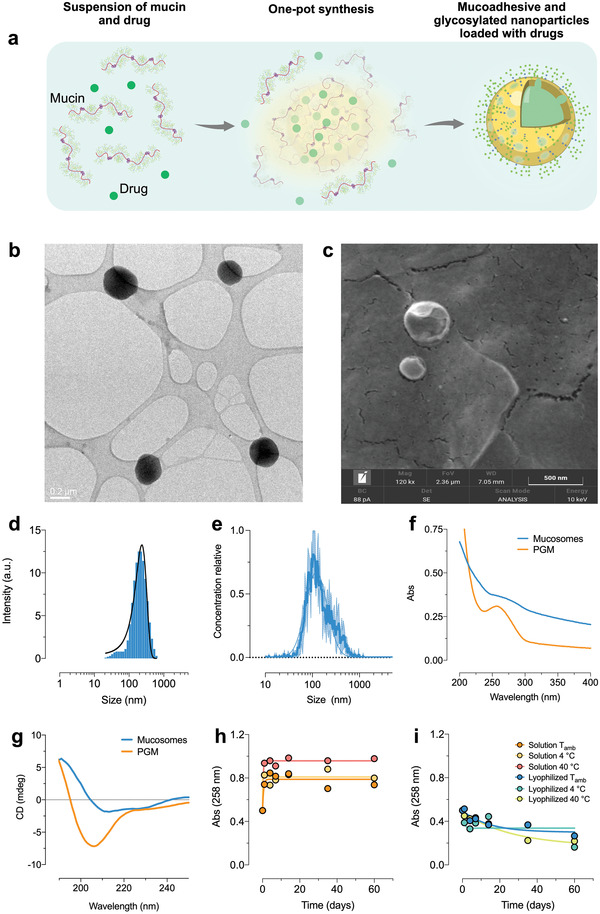
Mucosomes are nanoparticles of ≈200 nm, of spherical shape, and result stable when formulated as lyophilized powder. a) Mucin glycoprotein, which is the backbone of mucus, is used to synthesize mucosomes. The nanoparticles can be efficiently loaded with different compounds. The peptide core of mucin is highly glycosylated and glycans are preserved even after the formation of nanoparticles. b) Representative transmission electron microscopy images (scale bar = 0.2 µm) and c) field emission scanning electron microscopy analysis (scale bar = 0.5 µm) representing the size and the morphology of the mucosomes. d,e) Size distribution of mucosomes obtained by dynamic light scattering analysis and nanotracking analysis, respectively. f) Different UV–vis absorption spectra of native mucin glycoprotein (PGM) and mucosomes at the same concentration. g) Different circular dichroism spectra of PGM and mucosomes. h,i) The stability of mucosomes monitored by UV–vis spectroscopy was investigated at different temperatures and at two storage conditions: the absorbances of the lyophilized samples were more constant, especially in the first 20 d, with respect to samples stored as suspensions.

We, therefore, introduce a novel category of nanosystems, named *mucosomes*, which consist of glycosylated and mucoadhesive nanoparticles composed of mucins and which mirror the characteristics of mucus (**Figure** **1**a).

## 2 Results and Discussion

### 2.1 A Lean One‐Pot Synthesis of Stable Mucosomes

Proteins and other macromolecules can condensate in nanostructures through the desolvation process. Usually, organic solvent (e.g., alcohol, acetone) is added dropwise to an aqueous solution of protein under stirring to dehydrate the protein leading to protein self‐assembly and nanoparticle (NPs) formation.^[^ ^34^, ^35^ ^]^ Similarly to other proteins, native mucin is also described to form reversible nanoparticles.^[^ ^36^ ^]^ Building on state‐of‐the‐art knowledge, we set up a synthetic method to produce, in one single step, mucin‐based nanoparticles which both preserves the protein glycosylation and avoids the successive synthetic steps commonly employed to produce glycosylated nanoparticles.^[^ ^37^, ^38^ ^]^ The possibility to produce and load nanoparticles with drugs within the same synthetic procedure supports a lean preparation. The “produce‐and‐load” single step overcomes the limits deriving from successive loading and expands the diversity of the molecules that can be loaded as it is known that drug loading into pre‐formed nanoparticles is a challenge. Indeed, passive loading is an equilibrium process depending on the ratio between intraparticle and extraparticle volume, encapsulation efficiency is low and the formulation has to be purified from the nonencapsulated drug.^[^ ^39^ ^]^ Moreover, the encapsulation efficiency for high‐molecular‐weight molecules has been reported to be generally low, namely far below 50%.^[^ ^40^ ^]^ We, therefore, developed a production method based on desolvation, which allows simultaneous loading of drugs with encapsulation efficiencies spanning from 21% to 94% (see Section 2.8).

Stability in physiological conditions is another critical issue for an optimal applicability, along with the possibility of producing nanoparticles that can be stored until use, and/or resuspended in a medium without aggregation. To face these challenges, the desolvation method alone was not effective. The desolvation mechanism was paired with a secondary process, chemical crosslinking, and polyethylene glycol (PEG) was used to minimize aggregation. We called the novel class of protein nanoparticles, formed by the synergy protein desolvation with organic solvent and cross‐linkage with glutaraldehyde, “mucosomes.” Uncondensed mucin monomers and small mucin fragments were quantified in the supernatant volumes and the calculated yield of condensation is ≈25%. The formation of mucin nanoparticles can be appreciated when comparing the absorbance spectra of free mucin and mucosomes (Figure 1f): the characteristic band of porcine gastric mucin (PGM) at 258 nm, resulting mainly from the phenylalanine residues, strongly flattens after the formation of nanoparticles. Also, the scattering of the mucosomes is significantly higher because of the formation of nanoparticulate in suspension.^[^ ^41^ ^]^ The secondary structure of mucin (Figure 1g) strongly changes after the folding of the protein into nanoparticles as observed by circular dichroism. The predicted secondary structure of mucin varies from 29.2% *α*‐helix and 6.4% *β*‐strand, to 11.7% and 27.8%, respectively, representative of mucosomes.

Mucosomes are synthesized starting from commercially available porcine gastric mucin. The advantages of using the commercial protein are scalability, availability, and production costs. It should be acknowledged, however, that laboratory‐purified mucins may well be qualitatively superior and contain fewer impurities.^[^ ^42^, ^43^ ^]^

### 2.2 Morphological Characterization of Mucosomes

Key factors influencing their bioavailability of nanosystems are size and the morphology. The size and the morphology of mucosomes were evaluated by transmission electron microscopy (TEM), field‐emission scanning electron microscopy (FESEM), energy‐dispersive X‐ray analysis (EDX), dynamic light scattering analysis (DLS), and nanotracking analysis (NTA).

Mucosomes have a spherical shape with the size in the range of 150–300 nm (TEM analysis, Figure 1b). FESEM measurements (Figure 1c) corroborated the result (spherical nanoparticles of about 200 nm diameter). The observed nanoparticles are largely composed of C and to a lesser extent of O, N, and S (Figure S1, Supporting Information; EDX analysis). Such composition reflects the protein nature of the mucosomes.

By microscopy techniques, such as TEM and FESEM, samples are analyzed in a dried form. Additionally, as the physiological environment is relevant for their use, the size distribution profile in a liquid environment was evaluated by the DLS and NTA techniques (Figure 1d,e). The mucosomes exhibited a population of nanoparticles around 230 (±86) nm (DLS measurements), whereas the mean size values obtained by NTA are slightly smaller (170 ± 124 nm) than the measured size given by DLS. Even though only one population is observed with both techniques, the size distribution of mucosomes is moderately high. According to the polydispersity index (PDI 0.63 ± 0.13) and the span values (span 2.03 ± 0.21) it can be inferred that, within the same population, there is a wide range of size variation. A combination of high dilution factors for NTA and the different sizing principles of the two techniques is likely linked to the slight differences detected. Yet, both the results obtained in suspension agree with the size observed by TEM and FESEM analysis. Similar sizes (300 nm) are reported for a reversible nanosystem based on mucin, produced by glycerol‐induced condensation.^[^ ^36^ ^]^

Mucin is a negatively charged protein and, when assembled into mucosomes, nanoparticles acquire a negative charge. The repulsion of negatively charged NPs might prevent their agglomeration and maintain their stability. The zeta potential (ZP) of mucosomes, an indicator of the stability of colloidal dispersions, was measured by NTA (−20.8 ± 0.8 mV in phosphate buffer pH 7.4). Although mucosomes result with a negative ZP, the measured ZP is below the recognized minimum value necessary for colloids to be stable in solution (ZP > ±30 mV).^[^ ^44^ ^]^ For this reason, a resuspension step is needed immediately before use.

Mucosomes can be stored in suspension at different temperatures: at room temperature, 4 °C, and 40 *°*C. After an initial increase of the absorbance at 258 nm in the first day of storage, the values remained overall constant up to the longest test time: 60 d (Figure 1h). The sample stored at 40 *°*C presented the highest increase of absorbance. The increase of absorbance is linked to nanoparticle degradation as the disaggregation of mucosomes can release mucin in the solution. Following one of the approaches usually adopted to increase the long‐term stability of polymeric nanoparticles, mucosomes were lyophilized and the stability tests were repeated. Lyophilized mucosomes are more stable over time with respect to liquid formulations of the same, as the absorbances of the lyophilized samples are less subjected to variations over time when compared to the samples stored in suspension (Figure 1i). Mucosomes, therefore, can undergo lyophilization, an approach in line with the ones commonly adopted for other polymeric nanoparticles.

### 2.3 Mucosomes Are Glycosylated Nanoparticles

**Figure 2:**
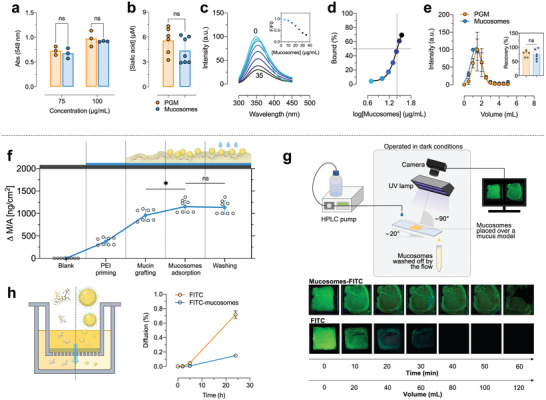
Mucosomes are glycosylated nanoparticles with mucoadhesive properties. a,b) The presence of carbohydrates on the surface of mucosomes is demonstrated by a Periodic acid‐Schiff (PAS) staining and by derivatization and fluorometric detection of sialic acid, respectively. The amount of carbohydrates is compared with the native protein. c) The interaction of mucosomes with Concanavalin A (Con A) was studied by steady‐state fluorescence spectroscopy and d) the equilibrium binding constant is calculated using a nonlinear fit. Interaction of mucosomes with Con A was also investigated by a chromatographic method. e) The elution volumes of PGM and mucosomes from a Sepharose column prepacked with Con A, while the inset shows the recovery of the injected amount of sample. f) Mucoadhesive properties of mucosomes were studied by QCM analysis. The mucosomes at first adsorb over the BSM‐PEI layer and remain adsorbed even after two washing cycles with PBS. g) The mucoadhesive properties were also investigated by a flow‐through assay by measuring the retention time of FITC‐loaded mucosomes on a cystic fibrosis mucus model. h) The mucopenetration of mucosomes was evaluated measuring the diffusion through a cystic fibrosis mucus model.

The mucin used to synthesize the mucosomes is a highly glycosylated protein, meaning that the peptide core of mucin is densely coated with sugars called glycans. The “sugar‐coating” of mucin gives it a huge water‐binding capacity and makes the protein resistant to proteolysis. The amount of glycans retained on mucosomes following the synthetic procedure was evaluated by a colorimetric periodic acid‐Schiff (PAS) staining of oxidized vicinal hydroxyls present on glycans. The amount of glycans detected on mucosomes is comparable to the amount present on the free protein, without statistical difference between the two samples (**Figure** **2**a). In addition to PAS staining, we also tested sialic acid groups, as a complementary proof of the surface glycosylation of mucosomes. Sialic acid is the general name for nine carbon acidic sugars with *N* or *O*‐substituted derivatives,^[^ ^45^ ^]^ widely distributed within the mucin structure, and has been shown to serve as a receptor for bacteria, such as *Pseudomonas aeruginosa*, and viruses, in the lower respiratory tract.^[^ ^46^, ^47^ ^]^ The sialic acid assay used was an improved Warren method,^[^ ^48^ ^]^ in which sialic acid is oxidized to formyl pyruvic acid. The latter reacts with thiobarbituric acid forming a pink‐colored product that can be detected by fluorometric detection (ex = 550 nm, em = 585 nm). The concentration of sialic acid found on mucosomes is comparable with levels found on mucin (Figure 2b). Taking together the results obtained from the PAS staining and the sialic acid assay, it is possible to conclude that the mucin glycosylation is maintained even after the synthetic process, and thus mucosomes could be considered glycosylated nanoparticles.

The mucosomes’ glycans retain their reactivity as it has been proven by studying the binding of mucosomes to a lectin glycoprotein known to bind mucin.^[^ ^49^ ^]^ Lectins are ubiquitous carbohydrate‐binding proteins of nonimmune origin. Among them, concanavalin A (Con A) is known to bind *α*‐glucosides, mannosides, and biopolymers with these sugar configurations. Previous studies have proved that Con A can bind mucin.^[^ ^50^ ^]^ Con A has an intensive fluorescence at 350 nm which is quenched upon the addition of increasing concentrations of mucosomes (Figure 2c). Based on the percentage of bound mucosomes to Con A, the estimated equilibrium concentration of mucosomes inducing half of the maximum binding was found to be 30.2 µg mL^−1^ (Figure 2d). The interaction with Con A was also confirmed by a chromatographic method: the eluted amount of mucosomes was quantified through a chromatographic column, prepacked with a Con A‐containing resin (Figure 2e). The retention volumes and the number of recovered samples of mucin and mucosomes were demonstrated to be comparable (PGM recovery 83 ± 17%, mucosomes recovery 77 ± 20%).

### 2.4 Mucosomes Are Mucoadhesive Nanoparticles

The possibility of nanocarriers to interact with mucus is a great added value since both the residence time and bioavailability of the encapsulated drug at the mucosal surface can be prolonged.^[^ ^33^, ^51^ ^]^ For instance, emerging intranasal applications are increasingly considered a valid option for local or systemic delivery of many therapeutic agents.^[^ ^52^ ^]^ Additionally, biodegradable and mucoadhesive polymeric carriers seem to be the most promising candidates for mucosal vaccine delivery.^[^ ^52^ ^]^ Although mucus turnover may impair the persistence of mucoadhesive nanoparticles it remains the case that the most challenging mucus‐related disorders, such as CF and chronic obstructive pulmonary disease (COPD), are generally characterized by an overproduction of mucus and a reduction (or loss) in the levels of mucus turnover. The permanent presence of bacteria participates in the inflammatory process contributing to a vicious cycle where mucus alteration, infection, and inflammation are tightly intertwined and difficult to separate. It is because of this complex pathological milieu that formulations which are able to increase the residence time of drugs within mucus are desirable as they prolong the therapeutic activity.

As a matter of fact, liposomes are consolidated and are the most widely used nanocarriers in the field of drug discovery. Liposomes are used for intracellular drug delivery, although they are not necessarily suitable to reach mucosal surfaces because of their lack of mucoadhesive properties. Only positively charged liposomes exhibit mucoadhesion, but, as a side effect, their biocompatibility is reduced.^[^ ^53^, ^54^, ^55^ ^]^ Different approaches to develop mucoadhesive liposomes include surface derivatization with other polymers (such as alginate, chitosan, pectin, Eudragit, and Carbopol) with processes that can increase the complexity of the synthesis and which impact on yield, costs, and additional purification and characterization steps.^[^ ^29^, ^56^, ^57^, ^58^, ^59^ ^]^

The hypothesis is that, since mucosomes are bioinspired from mucus and they are composed by mucin, they might be endowed with mucoadhesive properties. The molecular interaction of mucosomes with mucin was investigated by QCM‐D analysis. The test was studied at pH 7.4, the pH at which mucin (BSM) is negatively charged due to its isoelectric point around 3.^[^ ^60^ ^]^ The adsorption of mucosomes to a mucin layer, previously produced onto the quartz crystal, resulted in a resonance frequency shift of −25 ± 1 Hz which was calculated to correspond to a deposited mass of 196 ±10 ng cm^−2^. After the washing step, the frequency increased by 10% (2.5 ± 0.5 Hz), which corresponds to a mass loss of 20.5 ± 7 ng cm^−2^. The loss can be attributed to the removal of weakly bound NPs (Figure 2f). These analyses provide us with only a part of the whole picture of mucoadhesion. Therefore, we moved from a model at a molecular level to a model to the macroscale level. The mucoadhesivity of mucosomes on an in vitro cystic fibrosis mucus model was therefore evaluated by a fluorescent flow‐through assay. For this purpose, fluorescein isothiocyanate was encapsulated (encapsulation efficiency, 49 ± 12%) to obtain fluorescent mucosomes. It was observed that the retention time, expressed as wash out 50 (WO_50_), of fluorescein isothiocyanate (FITC)‐loaded mucosomes was almost 50‐fold higher than that of free FITC (49 vs 1 mL, Figure 2g). We also tested the ability of mucosomes to cross an in vitro tridimensional layer of mucus.^[^ ^61^ ^]^ The kinetics of diffusion of the FITC‐loaded mucosomes through the mucus layer was slower than that of the free dye for the whole duration of the test (up to 24 h) (Figure 2h).

Overall, these results suggest that dye‐loaded mucosomes have a longer residence time within mucus compared to the free dye. Longer persistence within mucosa could induce a higher drug bioavailability.

### 2.5 Cytotoxicity, Inflammatory Response, and Coagulation Effect of Mucosomes

**Figure 3:**
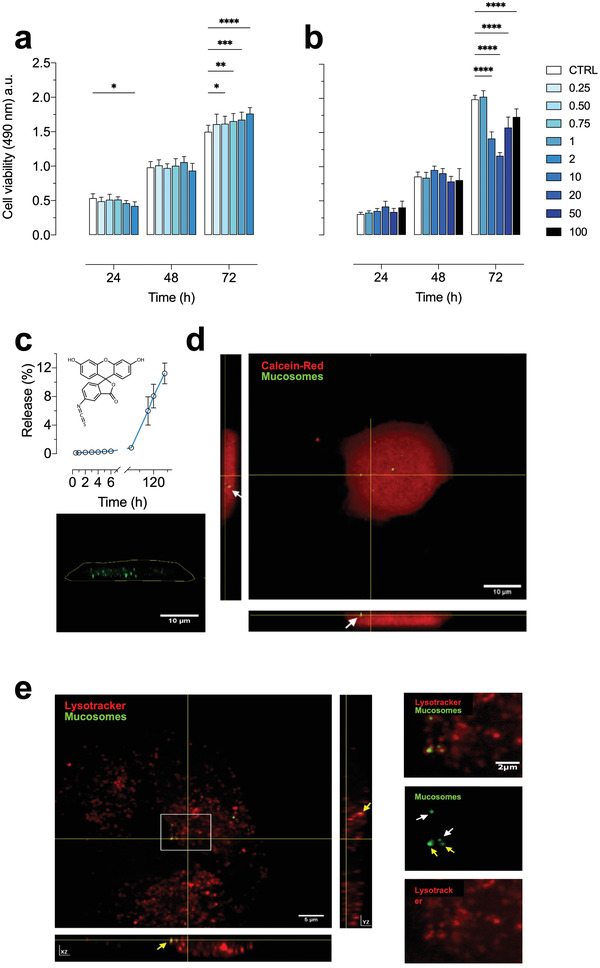
Mucosomes are cytocompatible on HeLa cells, internalized and partially colocalized with lysosomes. a,b) MTS cytotoxicity assay on HeLa cells after incubation with different concentrations (µg mL^−1^) of mucosomes and monitoring at different time points of a representative experiment. Data in the bar graph represent mean ± SD (*n* = 8 technical replicates). c) The release profile of FITC loaded into mucosomes investigated by dialysis. d) Confocal microscopy images of HeLa cells incubated with FITC‐loaded mucosomes. The red signal is referred to Calcein‐Red (ex: 561 nm); the green signal is referred to FITC‐loaded mucosomes (ex: 490 nm). The arrows on the sidebars point to internalized mucosomes. e) Intracellular localization of FITC‐loaded mucosomes. The red signal is referred to LysoTracker (ex: 561 nm); the green signal is referred to FITC‐loaded mucosomes (ex: 490 nm). The yellow arrows point out the mucosomes inside lysosomes; the white arrows point out the mucosomes outside the lysosomes. Orthogonal views are represented on the right and bottom sides. The white square represents the interested area which is magnified on the right showing from bottom to top: the LysoTracker; the FITC‐loaded mucosomes; the merged signal.

Mucosomes are well‐tolerated by HeLa cells cultured up to 72 h in presence of different concentrations of nanosystem (up to 2 µg mL^−1^) (**Figure** **3**a). The upper limit before cytotoxicity was found to be 10 µg mL^−1^, but only after 72 h of incubation (Figure 3b). These results are comparable or even less toxic than other organic nanosystems tested on the same cell line, such as poly‐caprolactone conjugated albumin nanoparticles^[^ ^62^ ^]^ and chitosan‐gold nanoparticles.^[^ ^63^ ^]^ Conversely, albumin nanoparticles and liposomes induce less cytotoxic effects when tested on HeLa cell line.^[^ ^62^, ^64^ ^]^

Nanocarriers able to cross the cellular membrane could be of interest if the aim would be to deliver active ingredients intracellularly. To isolate the signal of the nanoparticles around the cellular environment, mucosomes were loaded with FITC. However, before conducting the in vitro studies on cells, it was of fundamental importance to understand the release kinetics of the encapsulated dye. Thus, it is crucial to state with certainty that the signal recorded by confocal microscopy truly corresponds to the FITC loaded into mucosomes rather than to the released dye. For this purpose, the passive release of FITC was investigated by dialysis. A slow kinetics is observed in the first 24 h, followed by a step increase of release in the following days. The total amount released after the first 24 h is below 1%; after 7 d of monitoring 11% is released (Figure 3c). Given that the cellular localization tests are conducted within 24 h, we can suppose that the recorded emission of FITC corresponds to FITC‐loaded mucosomes. Results indicate that mucosomes are partially internalized and they colocalize with lysosomes (Figure 3d,e). In addition, cellular internalization was investigated using a quantitative approach. For this purpose, we used fluorescent‐activated cell sorting analysis (FACS) and it was revealed that ≈40% of the initial amount of FITC‐loaded mucosomes were internalized by HeLa cells after overnight incubation (Figure S3, Supporting Information). The specific internalization mechanism was not further investigated. Previous studies have shown a relationship between the size of nanoparticles and the endocytic pathway. Particles with a size below 200 nm were internalized into non‐phagocytic murine melanoma cells B16‐F10 via clathrin‐mediated endocytosis,^[^ ^65^, ^66^ ^]^ whereas particles larger than 500 nm have been known to enter phagocytic cells via phagocytosis pathways.^[^ ^67^ ^]^ Therefore, since mucosomes are nanoparticles of about 200 nm, they might be internalized by endocytosis.

**Figure 4:**
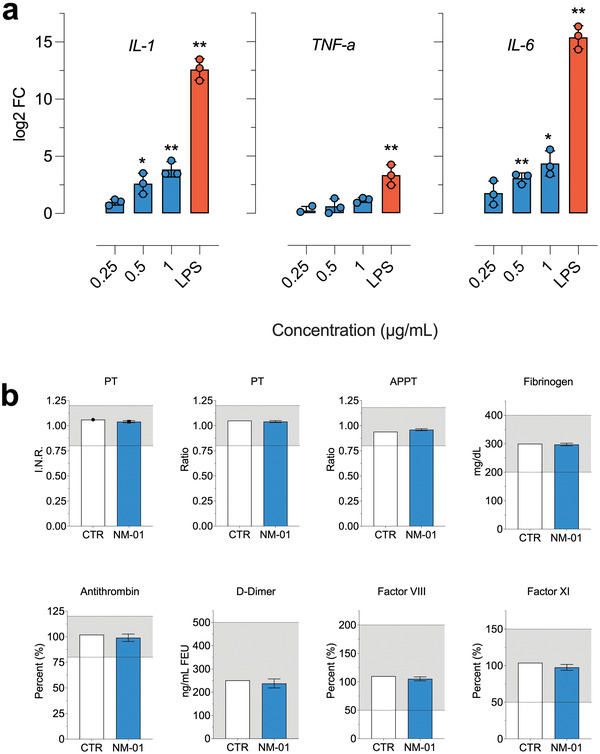
Mucosomes are not immunogenic and do not alter the coagulation cascade. a) IL‐1B, IL‐6, and TNF‐*α* mRNA expression on RAW 264.7 cells stimulated with different concentrations of mucosomes. LPS (1 µg mL^−1^) represents a positive control of an immunogenic agent. Log2 fold change (log2FC) expression was calculated using the −DDCt method using control untreated cells as a reference sample (0 value). 18S was used as a reference gene. The *X*‐axis represents genes selected for validation; *Y*‐axis represents normalized log2FC expression (mean ± corrected SD). * *p* < 0.05; ** *p* < 0.01. One sample *t*‐test against 0. b) Blood samples treated with mucosomes and the induced effect over several coagulation parameters.

Nanoparticles can exert important immunological effects such as immune cells interaction, inflammatory response triggering, complement cascade activation and antigenic‐specific hypersensitivity reactions. Immunotoxicological heterogeneity is a hallmark of nanomedicine and represents a critical hurdle for safety evaluations. The effect of mucosomes on RAW 264.7 cells, a cell line of mouse macrophages commonly used to assess inflammatory activity in vitro, was studied. The administration of exogenous materials could induce macrophages to secrete pro‐inflammatory cytokines, chemical messengers regulating the innate and adaptive immune system. The pro‐inflammatory response of the cells treated with mucosomes was analyzed by evaluating IL‐1B, IL‐6, and TNF‐*α* cytokines mRNA expression. The stimulation with mucosomes showed a minimal immune response as compared with the control, untreated, sample. In particular, although the transcription of proinflammatory cytokines did not significantly increase for the lower mucosomes concentration a significant increase in IL‐1B and IL‐6 was observed starting from 0.5 µg mL^−1^. Mucosomes, at any tested concentration, did not induce TNFa production. On the other hand, LPS (1 µg mL^−1^), which is known to activate antigen‐presenting cells, induced a strong increase in all of the three tests of cytokine transcription (**Figure** **4**a).

The intravenous route offers several advantages even when it comes to nanoparticle administration as it provides an almost instantaneous response and allows fine control of the drug concentration within the body. Blood is the first tissue nanoparticles encounter when administered intravenously and it is, therefore, of crucial importance to understand the biological response at this level. Several nanomaterials have been shown to alter blood coagulation pathways, producing unwanted side effects. For example, polystyrene nanoparticles activate intrinsic coagulation in a size‐dependent fashion^[^ ^67^ ^]^ and cationic polyamidoamine dendrimers induce platelet aggregation.^[^ ^68^, ^69^ ^]^ Another study reported that anionic liposomes shortened coagulation time in vitro and induced reversible aggregation of platelets both in vitro and in vivo through a factor XII‐ and XI‐mediated mechanism.^[^ ^70^, ^71^ ^]^ Mucosomes did not induce alterations of the coagulation cascade when the lyophilized mucosomes were added to human blood samples. Additionally, and more specifically, the prothrombin time (PT)—the activated partial prothrombin time (APPT) and the concentration of fibrinogen, antithrombin, D‐dimer, factor VIII, and factor XI—remained in the physiologically acceptable ranges and did not exhibit significant variations (Figure 4b).

### 2.6 The Potential of Mucosomes for Intranasal Drug Delivery

On the basis of demonstrated mucoadhesive properties it is reasonable to conclude that mucosomes could be a promising candidate for intranasal drug administration. The application of nebulizers for the pulmonary delivery of drug‐loaded mucoadhesive vehicles represents a noninvasive and portable mode of administration with high efficacy owing to optimal retention of the active ingredients at the target site.^[^ ^72^ ^]^

**Figure 5:**
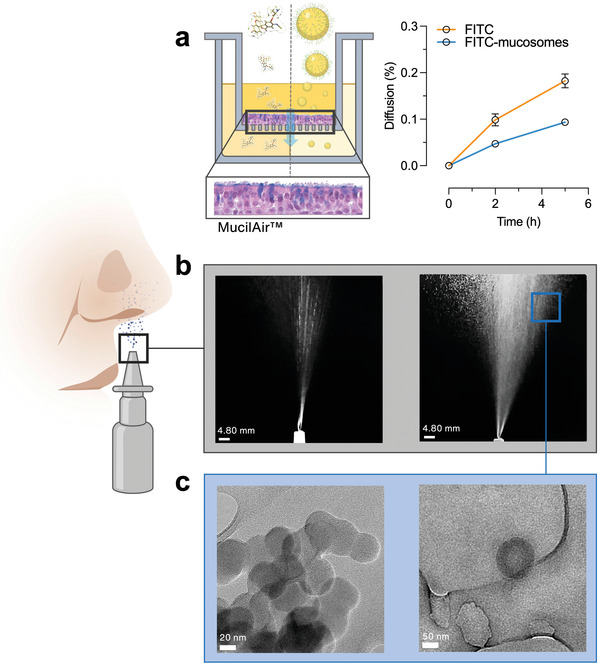
Mucosomes maintain shape integrity after spray nebulization and attach to the nasal epithelium. a) Mucosomes proved to increase the residence time of FITC within an in vitro cell model of the human airway epithelium. b) Schematic representation of the intranasal spray pump device and the spray geometry. c) Representative TEM images of the mucosome nanoparticles after nebulization through the generic intranasal spray pump device.

HeLa cells are a standardized cell line but far from modelling the airway epithelium. Therefore, diffusion studies of FITC‐loaded mucosomes were conducted on an in vitro reconstituted 3D human nasal epithelium (MucilAir), cultured at the air–liquid interface (**Figure** **5**a). After 5 h of incubation, the diffusion rate of the FITC delivered by mucosomes was slower when compared with FITC. Given that the MucilAir cell model is mucus producing and that mucosomes have proved remain for longer periods within mucus (Figure 2g) we can assume that the lower diffusion of FITC‐loaded mucosomes results from a longer residence time within mucus. When compared with the non‐cell‐based diffusion test (Figure 2g), the amount of diffused FITC through the cell model is higher. This can be explained: it has been previously proven that, in addition to transcellular and paracellular passive diffusion, fluorescein can also be actively transported.^[^ ^73^ ^]^ To ascertain the effect of dispensing mucosome‐based suspensions using nasal spray pumps, the shape stability of the inoculum was evaluated (Figure 5b). We were interested in evaluating the potential for shear induced nanoparticle degradation.^[^ ^74^ ^]^ The TEM analysis showed that after dispensing with a generic intranasal spray pump device, the integrity of mucosomes was not altered (Figure 5c).

### 2.7 Enzymatic Stability, Interaction with Serum and In Vivo Biodistribution Studies

**Figure 6:**
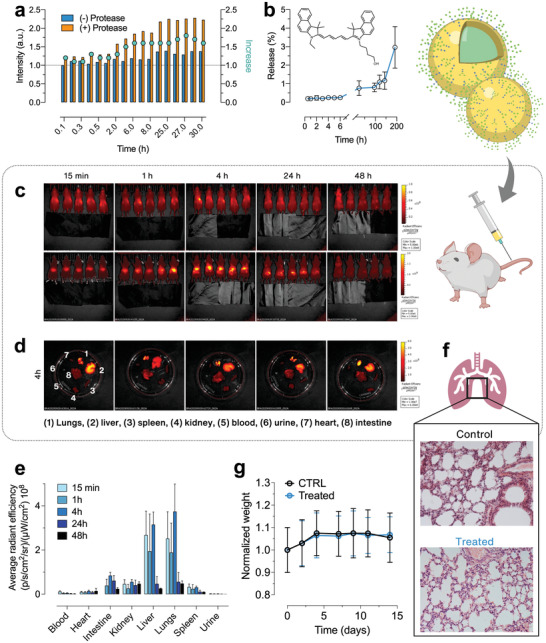
Protease accelerates molecular release from mucosomes. The mucosomes mainly distribute in the lungs and liver without inducing toxic effects. a) Stability of FITC‐loaded mucosomes in the presence of a generic protease. b) Release profile of Cy5.5‐loaded mucosomes studied by dialysis (MWCO 3.5 kDa). c) In vivo optical imaging of real‐time mice after administration of Cy5.5‐loaded mucosomes. d) Representative ex vivo optical imaging of mice organs sacrificed at the end of biodistribution tests. e) Quantitative fluorescence intensities of organs from ex vivo images. f) Histological analysis of Cy5.5‐loaded mucosomes in lungs of treated and untreated mice. The tissue slices were stained with hematoxylin and eosin (20×). g) Weight monitoring of treated and untreated mice after administration of mucosomes.

The biodistribution of novel nanoparticles is extremely important for the development of novel drug delivery systems. Once nanoparticles are introduced into the physiological environment, their biodistribution can be affected by many factors, including the chemical‐physical properties of nanoparticles, the characteristics of the environment and the route of administration. Before investigating the biodistribution of mucosomes, a stability assay of FITC‐loaded mucosomes incubated with a generic protease was conducted. The rationale behind such an experiment is to determine if mucosomes are sensitive to the activity of proteolytic enzymes that can be encountered after IV administration. If mucosomes are susceptible to protease activity, a higher release of the encapsulated compound is expected. Indeed, it was found that the fluorescence intensity of the dye increases over time in the presence of protease (**Figure** **6**a). This result suggests that mucosomes are sensitive to the proteolytic activity of the enzyme. After 30 h of incubation with the protease, the release of FITC is 1.6× higher than the release of FITC in absence of the enzyme (Figure 6a). However, even though this suggests/seems to indicate that mucosomes are enzyme‐susceptible it is worth mentioning that the experiment was conducted in a meticulously controlled environment, containing only the substrate (mucosomes) and the enzyme. The in vivo interaction might be less “aggressive” as the catalytic enzyme site might be subjected to endogenous competition. To better predict the in vivo performance of mucosomes, we analyzed their stability in serum over time.

After intravenous administration, the surface of mucosomes could associate with biomolecules from the surrounding environment forming the so‐called “biomolecular corona”. This phenomenon could increase the size of the particles as well as impact the distribution and the interaction with target sites (e.g., lectins). We observed that the size of mucosomes increases after 32 h of incubation with serum (133 ± 57 nm vs 308 ± 97 nm, Figure S2, Supporting Information), a result that can be attributed to the large serum proteins.^[^ ^75^ ^]^ The formation of the biomolecular corona is not necessarily associated with the hindering of the interaction of glycosylated nanoparticles with glycan‐binding biomolecules.^[^ ^76^ ^]^

To perform biodistribution studies, mucosomes were loaded with a fluorescent dye suitable for in vivo imaging tests. It is essential that the absorption and emission wavelength of the dye fall in a spectral region in which the interference with the biological matrix is minimal. For this purpose, a polymethine dye (cyanine 5.5 [Cy5.5]) was selected because it emits at 720 nm. In this spectral region the biological tissues are “transparent” and it is possible, therefore, to isolate and monitor only the signal coming from the fluorescent dye. Cy5.5 was encapsulated with an efficiency of 30 ± 8%. As it what was done with FITC, a release study of Cy5.5 was conducted to make sure that the signal recorded can truly be ascribed to the dye encapsulated within mucosomes. The release of Cy5.5 is below 1% even after 96 h, and remains below 5% after 200 h (Figure 6b). Subsequently, the in vivo biodistribution of Cy5.5‐loaded mucosomes was investigated after administering a dose of 400 µg mL^−1^ of mucosomes containing 13 nmol of Cy5.5 in the caudal vein of mice (Figure 6c). The biodistribution in lungs, liver, spleen, heart, and kidney was monitored up to 48 h. According to the slow release of Cy5.5 (Figure 6b), we can state that the monitored Cy5.5 signal corresponded to Cy5.5‐loaded mucosomes. An intense signal was observed at all the examined time points. Notably, in the first 4 h, the strongest signals were reported in the liver and lungs (Figure 6d) although significant fluorescent intensity also appeared in the kidneys and, interestingly, in the intestines (Figure 6e). After 24 h and, even more, at 48 h after injection, a weak signal was observed in all the monitored organs, indicating that mucosomes are gradually eliminated from 24 to 48 h. The biodistribution profile of mucosomes reflects a common accumulation pattern of nanoparticles of comparable size and nature. It has been reported that polymeric and spherical particles with a >150 nm diameter can readily accumulate within the lungs, liver, and spleen.^[^ ^77^, ^78^, ^79^ ^]^

In addition to the qualitative analysis, the amount of Cy5.5‐mucosome localized in some of the investigated organs was quantitatively measured. The lungs and the kidneys of one of the mice sacrificed one hour after the injection were randomly selected. The amount of Cy5.5‐mucosome present in the lungs represented 4.15% of the total administered dose, while at the same time point only 0.51% of the total dose was detected at the kidney level. These results agree with what was previously observed by optical microscopy. As the lungs have been demonstrated to be the organ with the highest distribution of mucosomes, it was interesting to evaluate if the presence of the nanoparticles could somehow affect tissue physiology. Thus, histology studies have been carried out on the explanted lungs. No signs of inflamed tissue were detected in the treated animals (Figure 6f).

Besides the assessment of biodistribution sites, the main goal of in vivo studies was to determine if mucosomes could function as a biocompatible nanocarrier. Studies have been conducted by injecting 100 µg of empty mucosomes (400 µg mL^−1^) intravenously into the caudal vein of five healthy mice. Injection of saline solution was administered into other five mice as control. The animals were weighed three times a week and monitored for clinical signs. Within the monitoring time (14 d), the animals remained healthy: no weight variations were observed and no abnormal clinical signs were manifested (Figure 6g). In the tested experimental conditions, up to 48 h, no adverse effect was observed in the mice.

### 2.8 Mucosomes Are a Versatile Drug Carrier

**Figure 7:**
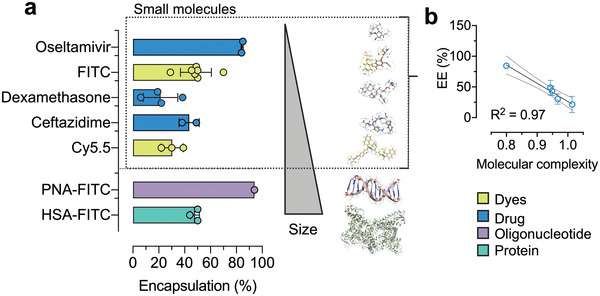
Mucosomes can be loaded with compounds spanning a wide range of molecular weights and physicochemical properties, and can increase residence time at mucosal surfaces. a) The entrapment efficiency of oseltamivir, fluorescein isothiocyanate (FITC), dexamethasone, ceftazidime, cyanine 5.5 (Cy5.5), peptide nucleic acid bioconjugated with FITC (PNA‐FITC), and human serum albumin bioconjugated with FITC (HSA‐FITC) was calculated based on the amount of free compound recovered from the washing volumes. b) Among the small molecules encapsulated, a good correlation was observed between entrapment efficiency (EE) and drug molecular complexity.

One of the most important characteristics of drug nanocarriers is their ability to be efficiently loaded with active ingredients. So far, mucosomes have been loaded with two fluorescent dyes, FITC and Cy5.5, with good encapsulation efficiencies. Given the physicochemical properties of mucosomes, they could be advantageous in pathological contexts characterized by conditions in which infection and inflammation are clearly intertwined. Drug delivery systems based on polymers are reported to improve the performance of existing antimicrobial compounds.^[^ ^80^, ^81^, ^82^, ^83^ ^]^ As a result, oseltamivir, ceftazidime, and dexamethasone have been selected as three models of antiviral, antibacterial and anti‐inflammatory drugs, respectively. Overall, a good encapsulation efficiency (EE) was observed (**Figure** **7**a). The antiviral oseltamivir is the drug with the highest EE (85 ± 0.7%), followed by the antimicrobial ceftazidime (44 ± 8%). On the other hand, the steroidal anti‐inflammatory dexamethasone is the least encapsulated (21 ± 13%). Deciphering the mechanisms governing the loading efficiency of drugs within mucosomes can be problematic. To find out if EE depends on any molecular property, we performed a correlation matrix with several molecular descriptors. To maximize the chemical variability, also FITC and Cy5.5 were included for the computation as they all belong to the class of small molecules (MW < 1 kDa). A negative correlation (*R* ^2^ = 0.97) between EE and drugs’ molecular complexity was found (Figure 7b). Molecular complexity is calculated by the number of distinct structural fragments, which one can construct from a molecule by just cutting parts off. The more distinct fragments there are, the more complex the molecule is.^[^ ^84^ ^]^ According to this relationship, it was found that higher entrapment efficiency corresponded with lower the molecular complexity.

In addition to interest in nanosystems carrying small molecules, there is now significantly increasing interest in the development of nanocarriers suitable for the delivery of macromolecules. Protein and nucleic‐acid based therapeutics have made important progress in the treatment of a variety of human diseases.^[^ ^85^, ^86^ ^]^ Given the high binding capability of mucins, we investigated if this property could be exploited to encapsulate macromolecules within mucosomes.^[^ ^27^, ^87^, ^88^, ^89^ ^]^ A small peptide nucleic acid (PNA) (TCACTAGATG, MW < 10 kDa),^[^ ^90^ ^]^ and human serum albumin (HSA; MW < 100 kDa) were selected as representative models of nucleic acids and proteins, respectively. Despite their high molecular weight, the two macromolecules showed excellent encapsulation efficiencies. Interestingly, the PNA decamer has been encapsulated with an efficiency of 94%. PNA is a synthetic analogue of DNA in which the ribose phosphate backbone has been replaced by a polyamide chain. Previous experimental and theoretical studies on the ionization and lipophilic properties of PNA derivatives have proven PNA to be a hydrophilic macromolecule even though the backbone is not charged like DNA and RNA.^[^ ^91^, ^92^ ^]^ Given that mucin is densely coated with carbohydrate chains, which represent strong hydrophilic domains, it is reasonable to speculate that PNA binds mostly on the glycosylated portions of mucin through an H‐bonding mechanism. Similarly, even though albumin is a high molecular weight protein (66.4 kDa), HSA was encapsulated into mucosomes with an efficiency of 48%. As albumin can form complexes with mucin,^[^ ^93^ ^]^ most probably by hydrophobic interactions,^[^ ^94^ ^]^ the overall high encapsulation into mucosomes can be explained.

## 3 Conclusion

In this study, we presented mucosomes, a novel nanosystem obtained through desolvation of mucin glycoproteins. We have been inspired by the unique properties of mucus, our first‐line of defence, to develop a cutting‐edge technology that exploits mucoadhesive and binding capacity. The synthesis, functionalization with glycans, and the loading with the desired active compound can just be completed in one single process. Mucosomes, spherical nanoparticles of about 200 nm, are stable over time when formulated as a lyophilized powder. The glycans present in mucin are preserved and still reactive on the surface of mucosomes. Mucosomes can encapsulate active ingredients spanning over a wide range of molecular weights. The in vitro biological tests demonstrate that mucosomes can reach the intracellular compartment without significant cytotoxic effects on HeLa cells. Mucosomes showed low immunogenicity as cytokine production on macrophages was minimal, when compared to a well‐known immunogenic agent (LPS), and proved to be inert over the coagulation cascade. In vivo tests showed that mucosomes have no adverse effects on mice and distribute mainly in the lungs and the liver. Within the monitored organs the concentration of mucosomes gradually decreased over time, suggesting that the nanoparticles do not lead to accumulation in tissues, a problem that usually can induce organ‐specific toxic effects.

The possibility to deliver active ingredients using mucosomes may offer several advantages over conventional systems in terms of mucoadhesive properties and targeted delivery, especially in pathological conditions where the mucus barrier represents an obstacle to effective treatment. The presence of surface glycans would mediate the engagement of glycoproteins expressed by pathogens such as bacteria and viruses. Drugs would be released more closely to pathogens, limiting their adverse effects, and maximizing their effectiveness. Given these unique features, we believe mucosomes can be a promising drug platform for mucosal delivery.

## 4 Experimental Section

### Materials

Mucin from porcine stomach (Type III, bound sialic acid 0.5%–1.5%, partially purified powder), mucin from bovine submaxillary gland (BSM), polyethylenimine hydrochloride (PEI), Schiff's reagent, sialic acid assay kit (MAK314), lectin from Concanavalin A Type IV, ready‐to use column prepacked with Con A Sepharose (HiTrap Con A 4B), glutaraldehyde 70% solution, polyethylene glycol (PEG) 6000, ethanol, sialic acid assay kit, alginate, d‐(+)‐Glucono‐delta‐lactone, CaCO_3_, oseltamivir, fluorescein isothiocyanate (FITC), Cy5.5, ceftazidime, dexamethasone, peptide nucleic acid, and human serum albumin were all obtained from Merck (Italy). Transwell permeable supports were purchased from Corning. All other reagents were of analytical grade and used as received. Millipore grade water (resistivity: 18.2 MΩ cm at 25 *°*C) was obtained from an in‐house Millipore system.

### Synthesis and Purification of Mucosomes

Mucosomes were prepared by a proprietary process (application PCT: PCT/IB2021/055450) using a desolvation technique. Initially, porcine gastric mucin (PGM) was suspended in 10 × 10^−3^ m NaCl to a final concentration of 25 mg mL^−1^. The suspension obtained was left under magnetic stirring for 1 h to homogenize the mixture. Then, the pH of the resulting opalescent suspension was adjusted with 1 m NaOH to pH 8.5–9. The suspension was left under magnetic stirring for 4 h. Ethanol was used as desolvation agent; it was added at a constant flow rate of 1 mL min^−1^ to reach a final PGM concentration of 5 mg mL^−1^. The addition of ethanol leads to protein desolvation and simultaneously the formation of protein nanoparticles. For the entire desolvation process, the suspension was kept under magnetic stirring. After the desolvation process, 8% glutaraldehyde was added to crosslink the nanoparticles. For each mg of PGM, 1.8 µL of glutaraldehyde was added and the mixture was left under magnetic stirring. To reduce particle aggregation, PEG 6000 was added to the mixture to obtain a final concentration of 2% (w/v). The suspension was kept overnight under magnetic stirring.

Mucosome purification was carried out by centrifugation of nanoparticle suspension at 10 000 RPM for 10 min at 4 °C. The supernatants were discarded and the sedimented nanoparticles were dispersed in mQ water using an ultrasound bath (1 min, room temperature, 45 kHz, 130 W). These purification steps were repeated five times. Eventually, the purified mucosomes were suspended and the concentration was calculated using a calibration curve. The yield of the synthesis is 30%–40%.

### Characterization of Mucosomes

The formation of mucosomes was characterized by UV–vis spectroscopy and the absorption spectrum was compared with that of the native protein. Both size and shape of mucosomes were monitored by TEM and FESEM. TEM samples were prepared by drop‐coating the mucosomes at a concentration of 0.1 mg mL^−1^ in water, into the carbon‐coated copper grid, and their size and morphology were characterized using a TEM (JEOL 3010‐UHR TEM operating at an accelerating voltage of 300.00 kV). The chemical elements present in the nanoparticles were evaluated through EDX. The FESEM sample was prepared at the same concentration and was analyzed using a FIB‐FESEM/EBSD//TOF‐SIMS Tescan S9000G.

The size distribution profile of mucosomes was determined by DLS (Malvern Zetasizer) and NTA (ZetaView – Particle Metrix). DLS measurements were performed after an equilibration time of 60 s which allowed samples to reach the temperature of 25 °C. A 5 mg mL^−1^ mucosome sample was diluted 1:50000 in ultrapure water. The sample was measured in size and zeta potential in scatter mode (488 nm laser). Triplicate measurements were performed for each sample.

The UV–vis absorption spectra were measured by a UH5300 Hitachi spectrophotometer at room temperature using a quartz cuvette (1 cm pathway length). Stability over time of mucosomes stored in suspension and as a lyophilized powder at different temperatures (*T*_amb_, 4 and 40 °C) was also evaluated by measuring the variation of the absorbance at 258 nm of samples (100 µg mL^−1^) at specific time points up to 60 d in comparison with the absorbance of freshly prepared samples. The shape integrity of mucosomes was also investigated after nebulization through an intranasal spray pump device. Mucosomes were suspended in water at a concentration of 1 mg mL^−1^ and nebulized using the spray device. The nebulized volume was collected and after 1:10 dilution with mQ water was analyzed by TEM microscopy as previously described. Suspensions of 0.05 mg mL^−1^ of PGM and mucosomes were scanned in the far‐UV spectral range over the wavelength region 190–250 nm with a scanning speed of 50 nm min^−1^ using a Jasco J‐815 spectropolarimeter equipped with a Xe arc lamp, using a quartz circular cuvette (path length 1 mm). CD spectra deconvolution and prediction of protein secondary structure were performed with K2D3 online software.^[^ ^95^ ^]^

### Drug Loading into Mucosomes

Oseltamivir, FITC, dexamethasone, ceftazidime, Cy5.5, PNA, and HSA were encapsulated within mucosomes. The drugs were solubilized into the mucin aqueous suspension prior to the desolvation step. The PNA was synthesized as previously described.^[^ ^90^ ^]^ Before encapsulation, HSA and PNA were bioconjugated with FITC following the reported protocol.^[^ ^96^ ^]^ The excess of FITC was 50‐fold the moles of HSA or PNA. After bioconjugation, the derivative was immediately purified using a Sephadex G‐25 desalting column and PBS (20 × 10^−3^ m, 150 × 10^−3^ m NaCl, pH 7.2) as eluent to evaluate the FITC labeling efficiency, the dye/protein ratio (D/P) of the conjugates was determined by the absorption spectra of the labeled HSA and PNA, registered in PBS, according to the relationship reported in Equation (1)^[^ ^97^ ^]^

(1) $$\frac{D}{P}=\frac{A_{\max}\varepsilon_{\mathrm{prot}}}{\left(A_{280}-cA_{\max}\right)\varepsilon_{\mathrm{dye}}}$$

where *A*_280_ is the absorbance of the conjugate at 280 nm; *A*_max_ is the absorbance of the conjugate at the maximum absorbance of the corresponding FITC; *c* is a correction factor that must be used to adjust the amount of *A*_280_ contributed by the dye because FITC also absorbs at 280 nm; *c* equals the *A*_280_ of the dye divided by *A*_max_ of the dye (*c* = 0.29); *ε*_prot_ and *ε*_dye_ are the molar extinction coefficients for the HSA or PNA and FITC, respectively.

Encapsulation efficiency was indirectly calculated by quantifying the drug released in the supernatants obtained throughout the purification steps. The released amount of FITC, Cy5.5, PNA‐FITC, and HSA‐FITC were detected by fluorescence spectroscopy using a Horiba Jobin Yvon Fluorolog 3 TCSPC fluorimeter equipped with a 450 W xenon lamp and a Humamatsu R928 photomultiplier. FITC and Cy5.5 were excited at 490 and 645 nm, respectively, while detection was measured at the maximum of fluorescence for each dye (*λ*_FITC_ = 520 nm, *λ*_Cy5.5_ = 715 nm). Oseltamivir, dexamethasone, and ceftazidime were quantified by HPLC‐MS/MS using a Varian HPLC equipped with a 410 autosampler and an Ascentis C18 column (10 cm × 2.1 mm, 3 µm), and detected on a Varian 320 MS TQ Mass Spectrometer equipped with an electrospray ionization (ESI) source operating in positive mode. The detector was used in multiple reaction monitoring (MRM) mode. The amount released was quantified based on linear calibration standard curves. The encapsulation efficiency was determined according to Equation (2)

(2) $$\mathrm{EE}\left(\%\right)=\frac{\mathrm{amount}\;\mathrm{of}\;\mathrm{drug}\;\mathrm{in}\;\mathrm{supernatant}\;\mathrm{volume}\left(\mathrm{mg}\right)}{\mathrm{initial}\;\mathrm{amount}\;\mathrm{of}\;\mathrm{drug}\left(\mathrm{mg}\right)}\times 100$$

Release studies were performed for the two dyes, FITC‐ and Cy5.5‐loaded mucosomes. The dye‐loaded mucosomes were suspended in a volume of mQ H_2_O to obtain a drug concentration of 1 mg mL^−1^. Drug‐loaded mucosomes (800 µL) were placed in dialysis tubes (MWCO 3500 Da), with an additional external volume of 29 mL of water, yielding a total volume of release medium of 30 mL. The release was performed at room temperature under magnetic stirring. At regular intervals, 1 mL samples were withdrawn and replaced with an equal volume of fresh water. The concentration of FITC and Cy5.5 was measured by fluorescence spectroscopy and quantified on seven‐point calibration curves as previously described. Experiments were repeated in triplicate.

The computational part was performed starting from the SMILES of the drugs and the dyes. The SMILES were retrieved from MarvinSketch (Marvin 20.20, 2020 ChemAxon) after drawing the molecular structure. Molecular properties were calculated with DataWarrior (ver. 5.5.0, openmolecules.org) and include physicochemical properties, drug‐likeness related properties, various atom and ring counts, molecular shape, complexity, flexibility as well as functional groups. The correlation matrix was calculated using DataWarrior.

### Diffusion of Loaded Mucosomes through Mucous Membranes

The diffusion of FITC‐loaded mucosomes was tested through a cystic fibrosis mucus model using Transwell permeable supports. The mucus model was prepared as described by Pacheco et al.^[^ ^61^ ^]^ Briefly, the cystic fibrosis mucus model was prepared using a modular approach mixing mucin from porcine stomach, alginate, CaCO_3_ suspension, and d‐(+)‐Glucono‐delta‐lactone solution. The mucus model (40 µL) was pipetted over the Transwell membrane. The donor compartments of the Transwell containing mucus model were then carefully shaken to uniformly distribute the mucus over the Transwell surface and to remove any air bubbles. Subsequently, the mucus model was left to crosslink overnight. Successively, FITC‐loaded mucosomes (200 µL of a 1 mg mL^−1^ suspension in mQ water) were inserted into the donor compartment of the Transwell plate, while 600 µL of mQ water were placed into the acceptor compartment. The donor and acceptor compartments were then incubated together. After 2 and 5 h, the entire acceptor volume was collected and replaced with fresh water.

Similarly, diffusion experiments were performed through an in vitro cell model of the human airway epithelium (MucilAir, Epithelix). MucilAir cell cultures were reconstituted from human primary basal, ciliated and goblet cells characterized by mucus production, active cilia beating, active ion transport, and tight junctions. Cells were cultivated, according to manufacturer instructions, using the recommended medium. Cells were cultivated at the air–liquid interface on Transwell inserts for one month before the experiments. Cells were treated with FITC or FITC‐loaded mucosomes (containing 0.6 µg mL^−1^ FITC) and donor and acceptor compartments were collected after 2 and 5 h analyses.

The amount of FITC diffused in the acceptor compartment was quantified by fluorescence spectroscopy as previously described in Section 2.4. The diffusion of the free FITC was used as a control.

### Assessment of Surface Glycosylation

To evaluate the glycosylation of mucosomes, a periodic acid assay was performed with periodic acid and Shiff's reactive. Briefly, the periodic acid oxidizes vicinal hydroxyls on sugars to aldehydes or ketones, which reacts with Schiff's reagent to give a magenta color. The intensity of the staining is proportional to the amount of glycans. Initially, PGM standards were prepared at 75 and 100 µg mL^−1^. In parallel, mucosomes were prepared at the same concentrations of PGM. Then, 10 µL of 50% w/v periodic acid was added to 7 mL of 7% v/v acetic acid. Subsequently, the PGM standards and mucosomes samples were incubated under agitation for 2 h and eventually centrifuged at 10 000 RPM for 45 min at 4 °C. At the end of centrifugation, the previously prepared solution of periodic acid (180 µL) was added to 600 µL of the supernatants. After 2 h of incubation at 37 °C, 60 µL of Schiff's reagent was added to each sample. The resulting solutions were kept in the dark for 30 min before measuring the UV–vis spectrum in a wavelength range of 400–700 nm.

In addition to the periodic acid assay, a complementary method was used to assess the surface glycosylation of mucosomes. The content of free sialic acid, which is one of the most common glycans present on mucins, was quantified. The sialic acid assay used was an improved Warren method,^[^ ^48^ ^]^ in which sialic acid is oxidized to formyl pyruvic acid. The latter reacts with thiobarbituric acid forming a pink‐colored product that can be detected by fluorometric detection. In brief, sialic acid standards for calibration curve were prepared by adding 5 µL of 10% trichloroacetic acid (TCA) to 20 µL of each standard. Similarly, 10 µL of 10% TCA were added to 40 µL of mucosomes at the concentration of 1 mg mL^−1^. Samples were vortexed and centrifuged at 14 000 RPM for 10 min, then 25 µL of the supernatants were transferred to clean tubes. Each sample was then oxidized and let stand at room temperature for 60 min. Afterward, the color reaction was initiated by adding 50 µL of a reactive dye and heating for 10 min at 100 °C. Samples were diluted 1:1 with DMSO, centrifuged at 14 000 RPM for 10 min and eventually sialic acid was quantified by fluorometric procedure (*λ*_ex_ = 550 nm, *λ*_em_ = 585 nm). The sialic acid concentration of each sample was calculated according to Equation (3)

(3) $$\begin{array}{ccc} \left[\mathrm{Sialic}\;\mathrm{acid}\right]\left(\mu M\right) & = & \frac{\mathrm{Fluorescence}\;\mathrm{sample}-\mathrm{Fluorescence}\;\mathrm{blank}}{\mathrm{Slope}\left(\mu M^{-1}\right)}\\ & & \times \;\mathrm{dilution}\;\mathrm{factor} \end{array}$$

### Interaction with Concanavalin A

The interaction between mucosomes and Con A was investigated by steady‐state fluorescence spectroscopy using a Horiba Jobin Yvon Fluorolog 3 TCSPC fluorimeter equipped with a 450‐W xenon lamp and a Humamatsu R928 photomultiplier. A constant concentration of Con A (10 µg mL^−1^) prepared in PBS was titrated with increasing concentrations of mucosomes (5, 10, 15, 20, 25, 30, 35 µg mL^−1^) prepared as previously described. Samples were excited at 280 nm and emission was recorded in the spectral range over the wavelength region 300–450 nm. Equilibrium association constant was obtained after a nonlinear fitting of the percentage of the bound mucosomes versus the concentration of mucosomes.

Interaction with Con A was also investigated using a 1 mL HiTrap Con A 4B Column. A 1 mg mL^−1^ suspension of mucosomes was injected into the ready‐to‐use column prepacked with Con A Sepharose 4B and 0.5 mL fractions were collected and analyzed by UV–vis spectroscopy. 20 × 10^−3^ m Tris‐HCl, 0.5 m NaCl, 1 × 10^−3^ m MnCl_2_, 1 × 10^−3^ m CaCl_2_, pH 7.4 was used as elution buffer. The same experiment was repeated with mucin, which was used as a reference. The amount of eluted mucosomes and mucin was monitored by measuring the absorbance at 258 nm, and the concentration was calculated using a calibration curve.

### Mucoadhesion

Mucoadhesion was studied by monitoring the interaction of mucosomes with mucin (BSM, mucin from bovine submaxillary glands, type I‐S) in real‐time using a dissipative quartz crystal microbalance QCM (QCM‐Z500, KSV Instruments, Finland). Briefly, QCM measures the change in frequency of an oscillating quartz crystal in response to the adsorption of material to the crystal surface. A mass deposited onto the surface of the crystal causes a decrease in its resonant frequency.^[^ ^98^ ^]^ The QCM response to viscoelastic layers was modeled using a Voigt model to calculate the deposited mass density.^[^ ^99^ ^]^ In this work, crystals having a diameter of 1.5 cm, a fundamental frequency of 5 MHz, and gold electrodes (100 nm and roughness of 0.9 ± 0.2 nm) were used. The crystal was mounted into a Teflon chamber having a volume of 2 mL.

As a first step, PEI (average MW 25 000 Da, *c* = 0.1 mg mL^−1^ in 0.01 m) was deposited onto the surface of the quartz crystal to impart a positive charge for the following deposition of mucin (*c* = 0.1 mg mL^−1^ in PBS 0.01 m). Namely, PEI and mucin were alternatively introduced into the chamber and left in contact with the crystal for 10 min – enough time to reach saturation adsorption for both molecules. After each deposition step, PBS 0.01 m was poured into the chamber and left in contact with the crystal for 1 min to remove the unabsorbed molecules. Finally, mucosomes were introduced into the chamber and left to interact with the mucin layer for 30 min, with the layer subjected to two washing steps of 15 min each. The frequency change of the crystal was continuously recorded during the experiments and data analysis was performed using the QCM Impedance Analysis software (KSV Instruments, version 3.11).

The mucoadhesion of mucosomes on an in vitro CF mucus model was also investigated via a fluorescence flow‐through assay developed by the Khutoryanskiy group.^[^ ^100^, ^101^ ^]^ Retention on the mucosal surface is dependent on the mucoadhesive properties of nanoparticles: higher adhesion rates to mucus correspond with longer times to wash out the nanoparticles and the higher washing out volumes. The cystic fibrosis mucus model was supplied by Bac3Gel Lda and carefully placed as 1.5 × 1.5 cm squared pieces over a microscope glass slide.^[^ ^61^ ^]^ Ammonium acetate 10 × 10^−3^ m, pH 6.6 was used to simulate the pH of the nasal fluid, and pumped at a constant flow of 2 mL min^−1^ over the glass slide using a Varian HPLC pump to model the washing off process of mucosomes.^[^ ^102^ ^]^ A UV–vis lamp fixed at 366 nm was placed at a distance of ≈25 cm and at ≈90° over the glass slide, ensuring homogeneous irradiation of the slide. A retention study was carried out by carefully spreading 150 µL of either 1 mg mL^−1^ FITC‐loaded mucosomes or 1 mg mL^−1^ FITC over the CF mucus layer. The slide was fixed at ≈20° from the ground to ensure constant flowing of the simulated nasal fluid. The mucus surface was captured using a camera placed at ≈90° over the glass slide. Each image was acquired in the same conditions of light intensity. The retention was monitored at regular time intervals by collecting the washed off sample; the amount of either FITC‐loaded mucosomes or FITC was quantified by fluorescence spectroscopy. Retention on the CF mucus model was quantified through WO_50_ values, which represent the volume of a biological fluid necessary to wash out 50% of a mucoadhesive formulation from a substrate.^[^ ^101^ ^]^ WO_50_ values of FITC‐loaded mucosomes and FITC were calculated via extrapolation of the wash‐off profiles to 50% using non‐linear fitting. The experimental setup is reported in Figure 2g.

### Cytotoxicity

The MTS assay was used to evaluate the cell viability of HeLa cells treated with mucosomes. Briefly, HeLa cells were seeded in each well of a 96‐well plate at a density of 2.5 × 10^3^ cells/well and cultured in a humidified 5% CO_2_ incubator at 37 °C for 24 h. The cells were then treated with mucosomes at various concentrations (0, 0.25, 0.5, 0.75, 1, 2, 10, 20, 50, 100 µg mL^−1^) in culture medium, and cells cultured without mucosomes acted as control. Then, MTS reagent was added to each well. The cells were further incubated for another 4 h. After incubation, the plate was shaken briefly and the optical density (OD) at 490 nm was measured. The cell viability was estimated according to Equation (4)

(4) $$\mathrm{Cell}\;\mathrm{viability}\left(\%\right)=\left[\frac{OD_{t}}{OD_{e}}\right]\times 100$$

where OD_e_ was the absorbance value estimated from cells without mucosomes and OD_t_ was the absorbance estimated in the presence of mucosomes.

### Cellular Uptake

Cellular uptake on HeLa cells of mucosomes was studied by confocal microscopy. For this purpose, mucosomes were loaded with FITC. The cells were seeded onto sterile culture dishes at a concentration of 2.5 × 10^5^ cells/well and cultured overnight (DMEM 10% FBS) prior to the experiments. After 24 h, the DMEM 10% FBS growth medium was aspirated and substituted with a growth medium containing 10 µg mL^−1^ of FITC‐loaded mucosomes. After having incubated culture dishes at 37 °C for 5 and 20 h, the modified growth medium was removed. The cells were treated with Calcein AM (CellTrace, calcein red‐orange, Molecular Probe, Life Technology) to obtain a red‐fluorescent cytoplasm. The calcein was diluted with Hanks’ Balanced Salt Solution (HBSS) to a 250 × 10^−9^ m concentration and then incubated for 30 min at 37 °C. Before the observations by confocal microscopy, the cells were washed twice with HBSS and fixed at 37 °C with a 4% paraformaldehyde (PFA) solution for 2 min. Images were acquired over the three‐axis of space (*x*, *y*, *z*) to reconstruct the entire cell volume. To visualize the samples by confocal laser scanning microscopy (CLSM) a DABCO MIX mounter was used. CLSM was performed with a TCS Leica SP8 X (Leica Microsystem) equipped with a scanner with DPSS laser (561 nm, to monitor calcein) and laser Ar (488 nm, to monitor fluorescein). The resulting images were obtained by an oil immersion lens (HC PLAPO CS2 63×/1.4 NA). The reconstruction of the 3D images helped to understand the uptake of the mucosomes. Images were analyzed with ImageJ software (Rasband, W. S., ImageJ, U.S. National Institutes of Health, Bethesda, MD, https://imagej.nih.gov/ij/, 1997– 2017). To assess the intracellular localization of mucosomes, HeLa cells were treated with 10 µg mL^−1^ of FITC‐loaded mucosomes. After treatment, the cells were incubated overnight and marked with Lysotracker‐Red (ex: 561 nm), a red fluorescent dye for labeling and tracking of acidic organelles in live cells (i.e., lysosomes). Fluorescence was monitored as previously described.

The cellular uptake was also investigated by FACS analysis. At first FITC‐loaded mucosomes were synthesized as previously described. HeLa cells were treated with FITC‐mucosomes overnight, then washed two times in PBS 1× and trypsinized. A total number of cells corresponding to 2 × 10^5^ were analyzed and 10 000 events were measured for each sample. All samples were acquired on a BD FACSVerse (BD Bioscience) and analyzed with FlowJO10.5.3.

### RNA Extraction and Quantitative Real‐Time PCR Analysis

The murine macrophage cell line, Raw 264.7, was used to study cytokine levels after stimulation with mucosomes. The cDNA levels of the pro‐inflammatory cytokines IL‐1B, IL‐6, and TNF‐*α* were tested by real‐time PCR technique. Cells were treated with mucosomes at three doses (0.25, 0.5, 1 µg mL^−1^). Untreated cells were used as a negative control while lipopolysaccharide (LPS 1 µg mL^−1^) was used as a positive control. Before the RNA extraction, the cells were observed by light microscopy to evaluate their viability and morphology.

RNA extraction and quantitative real‐time PCR (qRT‐PCR) analyses were performed as previously described.^[^ ^103^ ^]^ Briefly, total RNA was extracted using PureLink RNA Mini Kit (Thermo Fisher Scientific, Waltham, MA) and 0.5–1 *μ*g of total RNA were transcribed into complementary DNA (cDNA) by High‐Capacity cDNA Reverse Transcription Kit (Thermo Fisher Scientific, Waltham, MA). qRT‐PCR was performed using the following TaqMan Gene Expression Assays: Il1*β*, Mm00434228_m1; Il‐6, Mm00446190_m1; TNF*α*, Mm00443258_m1 (Thermo Fisher Scientific Waltham, MA). qRT‐PCR was performed on a QuantStudio 6 Flex Real‐Time PCR System (Thermo Fisher Scientific, Waltham, MA) and the analyses were done using QuantStudio Real‐Time PCR software. Transcript abundance, normalized to 18s messenger ribonucleic acid (mRNA) expression, is expressed as Log2 of the fold change over a calibrator sample.

### Assessment of the Effect of Mucosomes on the Coagulation System

The effect of mucosomes on blood coagulation was investigated in vitro. Blood samples were collected from healthy consenting volunteers who had not taken any medication in the 7 d before the tests. The test was carried out with nanoparticles in blood samples at a final concentration of 0.5 mg mL^−1^ and compared with samples not treated with mucosomes. After 30 min the addition of mucosomes, blood tubes were centrifuged at 2000 *g* for 15 min at room temperature to separate the corpuscular part from the plasma fraction. The PT, APPT, and the concentration of fibrinogen, antithrombin, D‐dimer, factor VIII, and factor XI were estimated by the automatic coagulometer ACL TOP 750 Las. Each sample was repeated in triplicate.

### Stability of Mucosomes to Protease and Serum

The stability toward a degradation enzyme of FITC‐loaded mucosomes was investigated using bovine pancreas protease from (Merck, Italy, code P4630). The protease degrades proteins by hydrolyzing the peptide bond. FITC‐loaded mucosomes were synthesized as previously described. A 1 mg mL^−1^ sample of FITC‐loaded mucosomes suspended in 10 × 10^−3^ m PBS, and a 0.01 mg mL^−1^ of protease prepared in the same buffer, were mixed in a 100:1 ratio. Afterward, the mixture was incubated at 37 °C. FITC‐loaded mucosomes without the protease were used as a control. At specific time points, 100 µL of the mucosome mixture was withdrawn and mixed with 83 µL of 110 × 10^−3^ m TCA, which was used to block the activity of the protease. The effect of the enzyme was measured by quantifying the amount of FITC released in the environment. FITC was detected by fluorescence spectroscopy using a Horiba Jobin Yvon Fluorolog3 TCSPC spectrofluorometer (ex: 490 nm).

The stability of mucosomes over time was also investigated in the presence of serum. Nanoparticle size was measured by DLS (Malvern Zetasizer) at 25 °C using a 1 mL sample volume. Each sample was measured three times. Samples for time course were prepared as 1:1 mixtures of 50 µg mL^−1^ mucosomes with 10% human serum (HS) suspended in water.

### In Vivo Biodistribution and Toxicity

The biodistribution of Cy5.5‐loaded mucosomes was investigated on healthy nude female Envigo mice via tail vein injection. The distribution profiles of Cy5.5‐loaded mucosomes in the heart, liver, spleen, lung, and kidney over different time periods (15 min, 1 h, 4 h, 24 h, 48 h) were comparatively investigated by fluorescence spectroscopy. The emission of Cy5.5 was monitored at 720 nm. A suspension of 400 µg mL^−1^ of Cy5.5‐loaded mucosomes was prepared in PBS and 250 µL (containing 13 nmol Cy5.5) were injected into mice (*n* = 10). Animals were randomly divided into two groups and 15 min, 1 h, 4 h, 24 h, and 48 h post‐injection mice were anesthetized with sevoflurane and monitored by optical imaging. After imaging, the mice were sacrificed and liver, spleen, kidney, lung, and heart were excised for ex vivo optical imaging acquisition. A drop of urine (where present) and blood were aspired and imaged as well. The analysis was performed with the Living Image IVIS software.

In addition to the qualitative analysis, Cy5.5 was also quantified in the lungs and kidneys of one of the mice. Organs were extracted, weighted, and suspended in 10 mL of ethyl acetate. Then, organs were homogenized using an Ultra‐Turrax T‐25 for 2 min and sonicated for another 2 min. To separate the corpuscular part from the solubilized material, the homogenate was centrifuged at 10 000 RPM for 10 min. The supernatant was collected and filtered through a 0.45 µm PTFE filter. Eventually, the Cy5.5 within the obtained solutions was quantified by fluorescence spectroscopy using a seven‐point calibration curve, as previously described.

Lung biopsies were obtained from treated and untreated mice. Tissue samples were fixed for 48 h in 4% w/v neutral buffered paraformaldehyde, dehydrated with gradient ethanol series, cleared in xylene, and embedded in paraffin. Sections (8 µm) were obtained using a Leitz microtome (Wetzlar, Germany) and were stained with hematoxylin and eosin (H&E). The slices were examined at 20× magnification under a light microscope (Zeiss Axiophot, Carl Zeiss, Jena, Germany), equipped with a digital camera.

Similarly, the in vivo toxicity of empty mucosomes was evaluated on the same animal model. A 400 µg mL^−1^ of mucosomes was prepared in PBS and 250 µL were injected into the caudal vein of healthy mice (*n* = 5). Saline was used as a control (*n* = 5). Animals were weighed three times a week and monitored for 14 d after administration. Mice were euthanized humanely two weeks after injection of empty mucosomes.

The studies were conducted according to the guidelines of the Declaration of Helsinki and were approved by the Institutional Review Board (or Ethics Committee) of the Italian Ministry of Health (Direzione Generale della sanità animale e dei farmaci veterinari).

### Statistical Analysis

All the quantitative data were reported as the mean ± standard deviation (SD). All the experiments in this study were carried out for at least three replicates for every group. ANOVA test and an unpaired *t*‐test were used to perform the comparisons for multiple groups and two groups, respectively. *p*‐values were deemed significant below *p* < 0.05, and indicated with asterisks (i.e., *p < 0.05, **p < 0.01, ***p < 0.001, and ****p < 0.0001). The Prism software package was used to perform the analysis.

## Conflict of Interest

The authors declare no conflict of interest.

## Supporting information

Supporting information

## Data Availability

The data that support the findings of this study are available from the corresponding author upon reasonable request.
