# Supplementary material for: Mucosomes: Intrinsically Mucoadhesive Glycosylated Mucin Nanoparticles as Multi‐Drug Delivery Platform
Source: Adv Healthc Mater. 2022 Jun 4;11(15):2200340. doi: 10.1002/adhm.202200340 (PMC11468529; doi:10.1002/adhm.202200340)
Supplement: Supplementary file 1 — Supporting information [file ADHM-11-2200340-s001.pdf]

ADVANCED HEALTHCARE MATERIALS

Supporting Information

for *Adv. Healthcare Mater.*, DOI 10.1002/adhm.202200340

Mucosomes: Intrinsically Mucoadhesive Glycosylated Mucin Nanoparticles as Multi-Drug Delivery Platform

*Cosmin Butnarasu, Paola Petrini, Francesco Bracotti, Livia Visai, Giuseppe Guagliano, Alessandra Fiorio Pla, Ettore Sansone, Sara Petrillo and Sonja Visentin**

Supplementary material

Mucosomes: intrinsically mucoadhesive glycosylated mucin nanoparticles as multi drug delivery platform

Cosmin Butnarasu, Paola Petrini, Francesco Bracotti, Livia Visai, Giuseppe Guagliano, Alessandra Fiorio Pla, Ettore Sansone, Sara Petrillo, and Sonja Visentin[*]

Cosmin Butnarasu, Francesco Bracotti, Sara Petrillo, Sonja Visentin
University of Turin, Department of Molecular Biotechnology and Health Science, via Quarello 15, 10135 Torino, Italy
[*]**Corresponding author email:**
sonja.visentin@unito.it

Summary

- **Figure S1.** EDX spectrum depicting the atomic composition of the mucosomes and the composition expressed as a percentage based on the EDX analysis (mean of n=3 independent batches).
- **Figure S2.** Effect of serum on mucosomes size measured by DLS analysis.
- **Figure S3**. Representative fluorescence-activated cell sorting (FACS) analysis of FITC-loaded mucosomes (green) and control (black) on HeLa cells. The inset depicts the percentage uptake of nanoparticles by cells. (n=4)

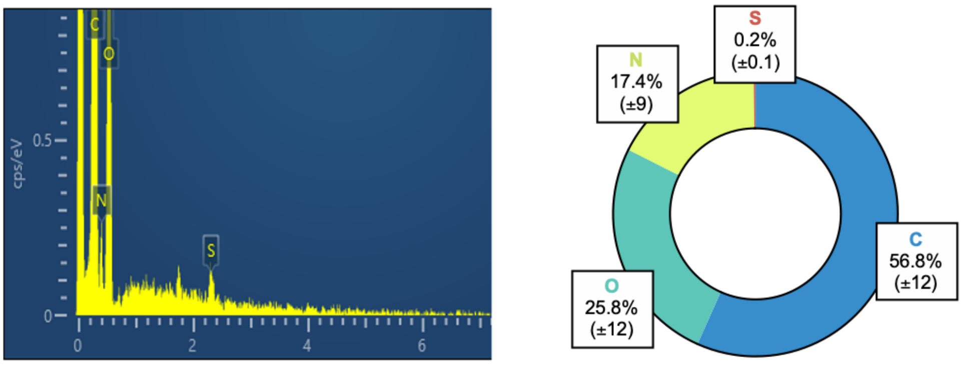

Figure S1. EDX spectrum depicting the atomic composition of the mucosomes and the composition expressed as a percentage based on the EDX analysis (mean of n=3 independent batches).

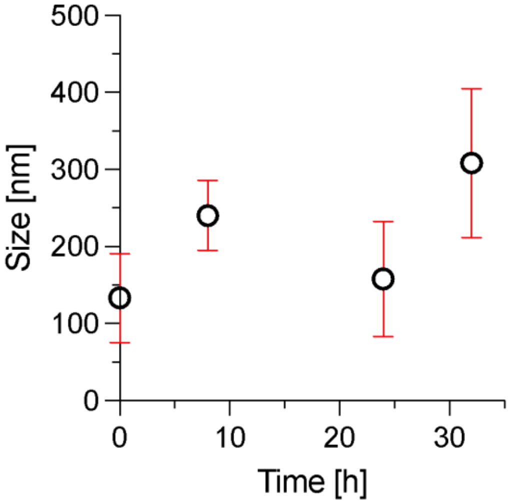

Figure S2. Effect of serum on mucosomes size measured by DLS analysis. Each sample was measured 3 times.

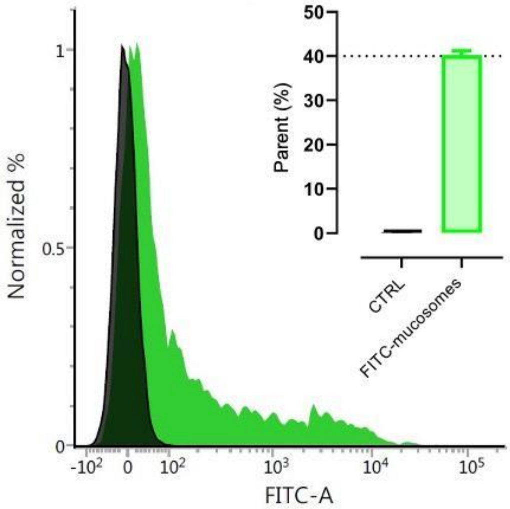

Figure S3. Representative fluorescence-activated cell sorting (FACS) analysis of FITC-loaded mucosomes (green) and control (black) on HeLa cells. The inset depicts the percentage uptake of nanoparticles by cells. (n=4)
